# 97 Comparison of Automated Antimicrobial Susceptibility Testing Instruments’ Results to Broth Microdilution Results

**DOI:** 10.1017/ash.2026.10519

**Published:** 2026-06-23

**Authors:** Elizabeth Monsees, Annie Wirtz, Jennifer Goldman, Adrienne Olney, Susan McElroy, Brian Lee

**Affiliations:** 1 Children’s Mercy Hospital; 2 Children’s Mercy Kansas City; 3 Children’s Mercy Hospitals and Clinics

## Abstract

**Background:** Traditional antimicrobial stewardship (AS) metrics focus on utilization and therapy optimization, but rarely capture the true impact on patient outcomes. Experience from safety initiatives, such as central line-associated bloodstream infections and serious safety events, has shown that metrics quantifying patient harm are essential to drive meaningful change. Related to AS, there is a need for novel measures to quantify antimicrobial-associated adverse drug events (ADE) to inform prescribing behavior. Prior research by infectious diseases (ID) experts found ADEs occurred in 21% of pediatric inpatient antibiotic courses, with risk increasing each day of therapy. Our objective was to assess harm events and identify opportunities for developing antimicrobial-associated harm measures to inform improvement efforts. **Methods:** We conducted a retrospective chart review to determine the incidence and classify antimicrobial-associated ADEs among hospitalized children. Three non-ID reviewers evaluated a random cohort of patients (<18 years) hospitalized ≤ 60 days between November 2019 and January 2020 who received oral or intravenous antimicrobials for ≥ 24 hours. Sampling was stratified by overall length of stay (LOS) tertiles (1-3, 4, 5+ days). ADEs were defined using clinical criteria from prior literature as occurring within 30 or 90 days of index hospitalization. Reviewers were trained by a nurse informaticist and an ID pharmacist prior to data collection. Weekly case reviews ensured consistency, with ID experts validating 10% of data for interrater reliability. ADEs were categorized by clinical type and antimicrobial class. The frequency of ADEs were compared across LOS category and patient-level total days of antimicrobial therapy (DOT). **Result:** Of the 411 patients evaluated, (median age: 4 years; 50.9% female) 16.8% (n=69) experienced ≥ 1 ADE. There were 78 total ADEs identified, mostly gastrointestinal (GI) (n=43, 55.1%), dermatologic (n=12, 15.4%), Clostridioides difficile infection (n=8, 10.3%), and vascular device-related events (n=7, 9.0%). GI ADEs were most often associated with fluroquinolones (n=3, 30%), penicillins (n=3, 22.6%), and 3rd generation cephalosporins (n=17, 13.8%). Median DOT was 3 [IQR: 1, 7]; differences in antimicrobial duration between patients with and without ADEs were considered non-significant (p <=/i< 0.2425). ADE incidence increased with LOS tertiles: 1-3 days (n=17, 11.6%), 4 days (n=21, 16.9%), and 5+ days (n=31, 22%). **Conclusion:** We demonstrated that antimicrobial-associated ADEs are frequent among pediatric inpatients. Developing standardized surveillance systems for antimicrobial ADE detection that are reliably incorporated into AS metrics may be a useful strategy to improve antimicrobial prescribing.

